# Open versus minimally invasive sacroiliac joint fusion: a multi-center comparison of perioperative measures and clinical outcomes

**DOI:** 10.1186/1750-1164-7-14

**Published:** 2013-10-30

**Authors:** Arnold Graham Smith, Robyn Capobianco, Daniel Cher, Leonard Rudolf, Donald Sachs, Mukund Gundanna, Jeffrey Kleiner, Milan G Mody, A Nick Shamie

**Affiliations:** 1 Jackville, FL, USA; 2 SI-BONE, Inc., 3055 Olin Ave, Suite 2200, San Jose, CA, USA; 3 Alice Peck Day Memorial Hospital, 129 Mascoma Street, Lebanon, NH 03766, USA; 4 Center for Spinal Stenosis and Neurologic Care, PO Box 8815, Lakeland, FL 33806, USA; 5 Brazos Spine, 1602 Rock Prarie Road, East Tower, Suite 2400, College Station, TX 77845, USA; 6 Medical Center of Aurora, Spine Center of Innovation, Suite 210, 1400 S Potomac St, Aurora, CO 80012, USA; 7 The Spine Clinic, 7925 Youree Drive, Suite 200, Shreveport, LA 71115, USA; 8 UCLA Spine Center, 1131 Wilshire Blvd, Suite 100, Santa Monica, CA 90401, USA

**Keywords:** Minimally invasive surgery, Sacroiliac joint, Arthrodesis, Open surgery

## Abstract

**Background:**

Sacroiliac (SI) joint pain is an under diagnosed source of low back pain due in part to lack of visible pathology on radiographs and symptoms mimicking other back-related disorders. Open SI joint fusion has been performed since the 1920s. This technique has fallen out of favor with the introduction of minimally invasive options. To date there has been no direct comparison between open and MIS SI joint fusion.

**Methods:**

We conducted a multi-center, retrospective comparative cohort study of patients who underwent SI joint fusion using either an open surgical (OS) technique using a combination of screws and cages or a minimally invasive surgical (MIS) technique with a series of titanium plasma spray (TPS) coated triangular implants. Operative measures including surgical operating time, length of hospitalization and estimated blood loss (EBL) were collected along with demographics and medical history, surgical complications, and 12- and 24-month pain scores. Improvements in pain were compared after matching for age and gender and controlling for a history of lumbar spine fusion using repeated measures analysis of variance.

**Results:**

Data were available for 263 patients treated by 7 surgeons; 149 patients treated with OS and 114 treated with MIS SI joint fusion. Compared to OS patients, MIS patients were on average 10 years older (mean age 57 vs. 46) and 69% of all patients were female. MIS operative measures of EBL, operating time and length of hospitalization were significantly lower than open surgery (p < 0.001). Pain relief, measured as change from baseline to 12 months in VAS pain rating, was 3.5 points lower in the MIS vs. OS group (-6.2 vs. -2.7 points, p < 0.001). When matched for age, gender and a history of prior lumbar spinal fusion, postoperative pain scores were on average 3.0 points (95% CI 2.1 – 4.0) lower in MIS vs. OS (rANOVA p < 0.001).

**Conclusions:**

In this multi-center comparative study, patients who underwent either OS or MIS SI joint fusion showed postoperative improvements in pain score. Compared to OS patients, patients who underwent MIS SI joint fusion had significantly greater pain relief and more favorable perioperative surgical measures.

## Background

Sacroiliac (SI) joint pain can be debilitating to patients, yet is an often-overlooked source of low back pain. Diagnosing the SI joint as the primary pain generator is difficult as patients often present with a combination of low back, groin, gluteal, and/or leg pain with signs mimicking radicular or discogenic distributions [1-3]. Furthermore, SI joint abnormalities may not be visible on imaging studies ordered to evaluate the lumbar spine.

The number of patients under-diagnosed and/or misdiagnosed is not inconsequential. Several studies report up to 30% prevalence of SI joint disorders in patients diagnosed with low back pain [2,4-6]. Disorders of the SI joint may be the result of trauma, pregnancy, inflammatory arthritis, osteoarthritis or degeneration of the joint either *de novo* or post lumbar spinal fusion [7]. SI joint pain after lumbar fusion is not uncommon. Two studies report prevalence rates of 40% and 43% [8,9]. Up to 75% of post-lumbar fusion patients develop significant radiographic SI joint degeneration after 5 years [10]. Diagnosis of SI joint disorders in the absence of acute trauma is made with a careful amalgamation of patient history, clinical exam, provocative physical tests, imaging, and diagnostic joint injections [11-14]. The treatment regimen often includes medication optimization, activity modification, physical therapy, therapeutic (i.e. steroid) joint injections and, in more severe cases, radiofrequency ablation [15]. For patients who do not experience adequate resolution of symptoms, surgical arthrodesis is an option.

Smith-Petersen and Rogers first reported SI joint arthrodesis in 1921 [16]. Studies that followed continued non-instrumented approaches to achieve arthrodesis and most required either long periods of immobilization or casting and bracing for a substantial period of time [17]. In the mid 1980s, reports of internal fixation using metal plates and screws began to appear [18-21]. Though casting and bracing were no longer required, perioperative morbidity was not trivial with relatively large incisions, significant bone harvesting, and lengthy hospital stays. Moreover, patients were kept non-weight bearing for several months postoperatively.

Reports of minimally invasive surgical (MIS) techniques to address the SI joint began appearing in 2008. However instrumentation remained limited to threaded screws and cages that rely on autologous bone graft [22-24]. Recently, there have been several reports of an MIS technique involving placing a series of triangular titanium implants across the SI joint (iFuse® Implant System, SI-BONE, Inc. San Jose, CA) with promising outcomes [25-29]. The design of this implant provides an interference fit into bone and the porous titanium plasma spray (TPS) coating on the surface allows for biological fixation. The confluence of these attributes renders the use of additional bone grafting material unnecessary.

According to a recent survey of spine surgeons, use of the MIS approach to fuse the SI joint has recently increased in popularity compared to traditional open fusion of the SI joint; in 2012, 85% of SI joint fusion was performed using minimally invasive techniques [30]. However, there have been no direct or indirect comparisons of these surgical methods. The purpose of this study is to compare operative measures, safety, and effectiveness between open surgical and minimally invasive SI joint fusion methods using a series of triangular titanium, porous TPS coated implants (iFuse Implant System, SI-BONE, Inc., San Jose, CA).

## Methods

A retrospective multi-center comparative study was undertaken after Institutional Review Board approval was obtained. Seven (7) surgeon sites participated; 3 surgeons who perform open SIJ fusion surgery (AGS, JK, MGM) and 4 who perform MIS SIJ fusion using triangular, titanium, TPS coated implants (LR, DS, MG, NS). A total of 263 patients were identified who underwent SIJ fusion surgery and had both pre-operative and 12- and/or 24-month postoperative pain scales documented in the medical chart; 149 in the open surgical technique cohort and 114 in the MIS technique cohort. Patients were treated between 1994 and 2012. Data extracted from the medical chart included demographics, history of prior lumbar spinal fusion, length of hospital stay (LOS), surgical operating time, estimated blood loss (EBL), complications of surgery and clinical outcomes using a 0-10 visual analog scale (VAS). Descriptive statistics are summarized as mean and standard deviation for continuous variables, and frequency charts for categorical variables.

### Diagnosis

All patients were diagnosed with SI joint disorders using a combination of detailed history, clinical exam, imaging and diagnostic injections. A positive result on 3 or more pain provocation tests such as Gaenslen’s, flexion abduction external rotation (FABER), compression, distraction and thigh thrust, was used as criteria for further testing to confirm the SI joint as the primary pain generator [11,31]. Diagnostic imaging studies such as x-ray, CT and MRI were performed on all patients to assess pathology in the lumbopelvic hip complex for differential diagnosis. Image-guided intraarticular anesthetic injections were performed as a final step to confirm the diagnosis. All patients had failed a 6-month course of non-surgical treatment consisting of a combination of medication optimization, activity modification, physical therapy and SI joint injections before they were offered surgery.

### Open technique overview

Several techniques for open fusion of the SI joint have been reported [7,20,21,24]. However, open SI joint surgery technique varied minimally between sites participating in this study. These variations are described where applicable. All sites in this study performed an open posterior approach to the SI joint. After the administration of general endotracheal anesthesia, the patient was placed prone on a radiolucent table, wired for electromyography, and prepped in the normal sterile fashion. A longitudinal incision was made centered over the posterior-superior iliac spine and deepened to expose the bone. Retractors were used to pull back the soft tissue and expose the posterior portion of the inferior SI joint. An osteotome was used to remove the portion of the posterior iliac crest that overhangs the SI joint. This bone was morselized and was later used as graft. Curettes and rongeurs were used to remove the cartilage from the articular portion of the joint and the interosseous ligament from the fibrous portion of the SI joint. One or two holes to accommodate cages were drilled into the SI joint and enlarged with a reamer. The cage(s) were packed with morselized bone and/or rhBMP (off label for this indication) and placed into position under fluoroscopic guidance. Additional bone material was then packed into the remaining open parts of the SI joint. Two 6.5 × 40 mm cancellous iliosacral lag screws were then placed in standard fashion. Under fluoroscopic guidance, pins were placed from lateral to medial across the ilium, across the SI joint and into the sacrum. The pins were then over drilled with a cannulated drill. The holes were tapped with a 6.5 mm tap and the screws with large washers were placed. At one site (AGS), the fixation consisted of a 6.5 mm pedicle screw placed into the S1 pedicle and a second 6.5 mm pedicle screw placed between the inner and outer tables of the ilium. A 3 cm spinal rod was then used to connect the two screws. As for the fixation, one surgeon (MGM) used recon plating across the SI joint with placed 2 cancellous screws placed in both the sacral ala and the ilium, as well as one long percutaneous cannulated screws across the SI joint. For all surgeons, EMG stimulation was used throughout the procedure to ensure safe placement of instrumentation. The wound was then irrigated, a hemovac drain was placed between the deep and superficial fascia, and the tissue layers were closed sequentially.

### MIS technique overview

**Figure 1:**
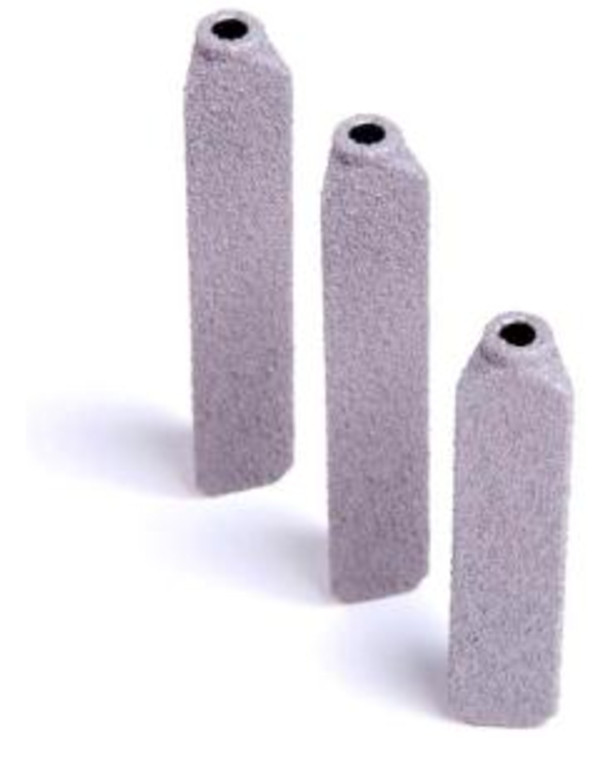
iFuse Implant System

**Figure 2:**
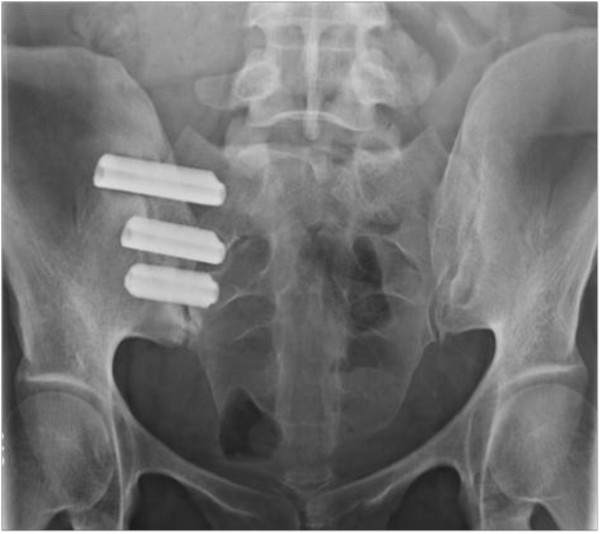
Postoperative radiograph demonstrating placement of 3 implants.

Minimally invasive SI joint surgery using a series of triangular, titanium, TPS coated implants (iFuse Implant System) (Figure 1) was performed with the patient on a radiolucent table to facilitate the use of intraoperative fluoroscopy. After general endotracheal anesthesia was administered, the patient was turned prone and prepped in the normal sterile fashion. A lateral incision (3 cm) was made into the gluteal region, positioned over the sacral body as viewed on a lateral fluoroscopic image. The fascia was then bluntly dissected to reach the outer table of the ilium. A Steinmann pin was passed through the ilium across the SI joint to the center of the sacrum (lateral to the neural foramen). After a soft tissue protector was passed over the pin, a hand drill was used to create a pathway across the ilium, across the SI joint and into the sacrum. Finally, a triangular broach was used to further decorticate the bone and prepare a triangular channel to receive the first implant. Using a pin guidance system, a total of three implants were placed. The most cephalad implant was seated within the sacral ala above the first neural foramen. The second implant was located above or adjacent to the S1 foramen and the third between the S1 and S2 foramen (Figure 2). The incision was irrigated and the tissue layers were closed. A variable program of gradual return to full weight bearing was employed based on local practices and patient needs. In general, patients were instructed to ambulate partial weight bearing with the assistance of a walker for the first 3 weeks after which time toe touch ambulation was recommended for another 4 weeks. After a regimen of gradual return to full weight bearing, patients began 4 weeks of physical therapy.

### Clinical outcome assessments

Patient reported clinical outcomes were collected prospectively prior to surgery to establish baseline values and at follow up intervals per each surgeon’s routine practice. For consistency in reporting, pain scores using visual analog scale (VAS) were collected at visits close to 12 and 24 months only.

Clinical improvement was defined using minimum clinically important difference (MCID) and substantial clinical benefit (SCB) values available in the literature. As there are currently no reported MCID or SCB values for SI joint fusion, values were chosen using lumbar spine criteria published by Copay et al. for MCID and Glassman et al. for SCB [32,33]. MCID for pain is defined as a change of >2.0 pts and SCB is defined as a 2.5-point decrease or raw score of < 3.5.

### Statistical analysis

Baseline demographic variables were summarized with mean, standard deviation and frequency tables. Demographic characteristics were compared across cohorts using t tests for continuous variables and chi-squared tests for nominal variables. Surgical parameters (e.g., operating time, estimated blood loss, length of stay) were compared using t tests.

A key outcome variable was the change in pain scores on VAS. Mean baseline VAS scores, as well as mean change from baseline at 12 and 24 months, were tabulated. To account for potential differences in patient characteristics across cohorts, VAS pain scores were compared after matching, as follows. Patients in the two treatment groups were matched based on gender and age in 5-year intervals (20–25 years, 25–30 years, etc.). This process led to a variable m:n matching where m ≠ n. Variable matching was due in part to missing values of the pain score as well as differences in overall age distribution between the two cohorts.

A linear mixed effects model (SAS PROC MIXED) was used to estimate treatment differences using a cell means formulation with additional covariates of prior lumbar fusion and a random effect for patient. Prior lumbar fusion was included as a covariate since it may increase the risk of SI joint degeneration, and also reflects a history of serious back pain requiring surgery. The cell means formulation has a parameter for each treatment, each block (i.e., gender and 5-year age category), visit, and a random effect for patient. Pairwise differences between treatments for blocks of matched age-category/gender were estimated. Sparse combinations of age/gender blocks that resulted in inability of the model to provide estimates were removed from the model. The final model was estimable and converged. A final treatment effect estimate was calculated by averaging over matched age-category/gender/treatment combinations. Analysis was performed using SAS 9.0 (Cary, NC).

## Results

A total of 263 patients were identified; 149 patients treated with open surgery (OS) and 114 patients who underwent minimally invasive surgery (MIS). Demographic characteristics of patients are shown in Table 1. Patients undergoing open surgical fusion were younger (mean age 45.8 vs. 57.4 years) and were less likely to have had prior lumbar fusion (23.5% vs. 47.4%). Approximately 70% of all patients, regardless of surgery type, were women. Bilateral surgery was more common in those undergoing minimally invasive SI joint fusion.

**Table 1:** Patient demographics

	**Open surgical fusion**	**Minimally invasive fusion**	**P-value**
N	149	114	-
Age, mean (SD)	45.8 (11.3)	57.4 (14.0)	<.0001
Female, n (%)	103 (69.1%)	82 (71.9%)	0.6220
Prior lumbar spine surgery, n (%)	35 (23.5%)	54 (47.4%)	<.0001
Side treated			
Left	74 (49.7%)	41 (36.0%)	0.0120
Right	71 (47.7%)	62 (54.4%)	
Both	4 (2.7%)	11 (9.6%)	

Peri-operative measures were overall lower in the MIS group (Table 2). Open surgical fusion was a longer surgery, requiring an average (±SD) 163 ± 25 minutes compared to MIS fusion, which took just over an hour (mean 70 ± 24 minutes). Although blood loss estimates were available in only 60% of MIS patients, mean estimated blood loss was approximately one-eighth that of open SI joint fusion (288 ± 182 cc in OS, 33 ± 27 cc in MIS). Most patients who undergo MIS SI joint fusion stay in the hospital overnight or are discharged the same day. Hospital length of stay, available from only 1 surgeon in the MIS cohort (MG) and all patients in the open cohort, was substantially shorter in the MIS group (mean 1.3 days) compared to the OS group (mean 5.1 days).

**Table 2:** Operative Measures

	**Open surgical**		**Minimally invasive**		**P-value**
		**Fusion**		**Fusion**	
	N	Mean (SD)	N	Mean (SD)	
Operating room time	100	163 (25)	63	70 (24)	<.0001
Estimated blood loss	138	288 (182)	66	33 (27)	<.0001
Hospital length of stay	137	5.1 (1.9)	30	1.3 (0.5)	<.0001

Mean baseline VAS pain scores were just over 1 point higher in the MIS vs. open groups (8.3 vs. 7.1, p < .0001) (Table 3). At follow-up, the raw improvements from baseline in VAS pain scores were 3.6 and 3.7 points lower in the MIS group compared to the open group at 12 and 24 months after surgery, respectively. Using age and gender-matched blocks, repeated measures analysis of variance that also controlled for a history of prior lumbar fusion showed that the adjusted mean VAS pain scores during follow-up were 3.02 points lower in MIS vs. open (p < .0001, 95% CI 2.07-3.99 points). The proportions of subjects showing a 2 or greater point decrease in pain scores at follow-up compared to baseline were 86% and 82% in the MIS group at 12 and 24 months, respectively, vs. 61% and 50% in the open group at 12 and 24 months (Table 4). Substantial clinical benefit was reached for 58% and 47% of patients in the open fusion group at 12 and 24 months, respectively. The percentage of patients reaching SCB in the MIS group was higher at 86% and 82% (Table 3). Decreases in pain scores were larger in the MIS group compared to the open group amongst patients either with or without a history of prior lumbar fusion, a known risk factor for SI joint degeneration (Table 5). A small number of patients underwent bilateral SI joint fusion (OS 4 cases, MIS 11 cases); a comparison of clinical outcomes between these groups could not be performed due to the small sample sizes.

**Table 3:** SI joint pain ratings at baseline and at 12- and 24-months postoperatively

	** Open surgical**		**Minimally invasive**	
	**Fusion**		**Fusion**	
	**N**	**Mean (SD) or %**	**N**	**Mean (SD) or %**
VAS pain score at baseline	139	7.1 (1.9)	113	8.3 (1.6)
VAS pain score at 12 months	114	4.6 (3.0)	94	2.3 (2.6)
VAS pain score at 24 months	58	5.6 (2.9)	38	1.7 (2.9)
Change in VAS pain score at 12 months	113	-2.7 (3.2)	93	-6.2 (3.1)
Change in VAS pain score at 24 months	58	-2.0 (3.3)	38	-5.6 (3.5)

**Table 4:** Improvements in SI joint pain at 12- and 24- months post-operatively

	**Open surgical**		**Minimally invasive**	
	**Fusion**		**Fusion**	
**Change Category**	**12 Mo**	**24 Mo**	**12 Mo**	**24 Mo**
Improvement of at least 2 points	69 (61.1%)	29 (50.0%)	80 (86.0%)	31 (81.6%)
Improvement of <2 points	17 (15.0%)	7 (12.1%)	6 (6.5%)	1 (2.6%)
No change or worsening	27 (23.9%)	22 (37.9%)	7 (7.5%)	6 (15.8%)
Improvement of at least 2.5 points or postoperative score <3.5 points	66/114 (58%)	27/58 (47%)	81/94 (86%)	31/38 (82%)

**Table 5:** SI joint pain ratings by history of prior lumbar spinal fusion

	**Open surgical fusion**				**Minimally invasive fusion**			
	**Prior lumbar fusion**				**Prior lumbar fusion**			
	**Yes**		**No**		**Yes**		**No**	
	**N**	**Mean (SD)**	**N**	**Mean (SD)**	**N**	**Mean (SD)**	**N**	**Mean (SD)**
VAS pain score at baseline	33	7.0 (1.7)	106	7.2 (1.9)	53	8.5 (1.3)	60	8.1 (1.9)
VAS pain score at 12 months	30	5.4 (3.0)	84	4.4 (2.9)	51	3.0 (2.9)	43	1.5 (1.9)
VAS pain score at 24 months	10	6.7 (2.0)	48	5.4 (3.1)	17	1.9 (3.2)	21	1.5 (2.6)
Change in VAS pain score at 12 months	29	-1.8 (3.1)	84	-3.0 (3.2)	50	-5.5 (3.2)	43	-7.0 (2.8)
Change in VAS pain score at 24 months	10	-1.5 (1.9)	48	-2.1 (3.5)	17	-6.0 (3.7)	21	-5.2 (3.4)

### Complications

No intraoperative complications occurred. Postoperative complications were slightly more common in the open surgery group (21% of patients) compared to the MIS group (18%) (Table 6). The most common complications reported in both groups were postoperative neuropathy and transient trochanteric bursitis (4 OS and 2 MIS). In the OS group, leg pain (3), neuropathy (4) and wound related issues (6) were more common. In the MIS group, falls (4) and facet pain (4) were more frequent.

**Table 6:** Postoperative adverse events

**Adverse events**	**OS**	**MIS**
Bone fragment near upper sacral screw causing pain	1	0
Buttock hematoma	0	2
Cellulitis	1	3
Deep venous thrombosis	1	0
Facet pain	0	4
Fall	2	4
Hip pain requiring spinal cord stimulator	1	0
Iliotibial band pain	2	0
Leg pain	3	0
Lipoma in wound scar requiring surgical removal	1	0
Low back pain	0	1
Painful heterotopic ossification	2	0
Piriformis syndrome	0	2
Pneumothorax	1	0
Postoperative neuropathy	4	0
Pulmonary embolism	2	0
Scar pain requiring block	1	0
Screw loosening	1	0
Screw replacement misplacement	1	0
Strained buttock muscle	0	1
Trochanteric bursitis	4	2
Wound dehiscence	1	0
Wound infection	3	1
Wound seroma	2	0
Total	34	20

Forty-four percent (66/149) of patients in the open surgery cohort subsequently underwent removal of spinal implants. In most cases, removal was for pain at the ilial or sacral screw. In contrast, only 3.5% of patients (4/114) undergoing MIS surgery underwent postoperative repositioning of the implants. In 3 patients, reoperation was due to nerve root impingement discovered on the postoperative CT scan. In one case, reoperation was performed at the surgeon’s discretion based on radiographic findings only.

## Discussion

Confidently diagnosing the SI joint as a pain generator is a challenging endeavor as symptoms may mimic other conditions such as lumbar and hip pathology. A history of sleep disturbance, pain on prolonged sitting and leg instability, pain in the low back, buttock, hip and groin as well as the SI joint are common. Furthermore, imaging studies ordered to evaluate low back or hip pain typically do not include a clear view of the SI joint. Unless educated to examine the SI joint, an improper diagnosis may be made. An accurate diagnosis requires a combination of history, physical examination maneuvers that stress the SI joint, and image-guided diagnostic injections into the joint.

Multiple surgical and non-surgical treatments for SI joint disorders are available. When non-surgical management fails to provide adequate relief of symptoms, surgical stabilization is an option. Published case series of various arthrodesis techniques report variable improvements in pain and function with more invasive approaches reporting moderately high complications and non-unions (Table 7) [17,19-24,26,27,29,34,35]. MIS techniques that use internal fixation, such as cages, plates and screws, often rely on bone graft harvesting, which may negatively affect outcomes.

**Table 7:** Reports of SI joint fusion

**Author, Year**	**N**	**Demographics**	**Diagnostic Standard**	**Surgical Procedure/Post-op care**	**Results**	**Complications**
Rudolf, 2012 [27]	50	Age: 54	3 or more positive	iFuse Implant System	OR time: 65 +/- 26 min	Superficial cellulitis: 3
		Gender: 34 F/16 M	provocative maneuvers, confirmatory joint		Mean improvement on VAS: -4.3 pts at 12 months	Deep wound infection:1
		Prior lumbar fusion: 44%	injections		Satisfaction 82% at 12 months	Hematoma: 2
						Reoperation: 3
Sachs, 2013 [26]	40	Age: 58	3 or more positive provocative maneuvers, confirmatory joint injections	iFuse Implant System	Mean improvement on VAS of -7.8 pts (p < 0.001)	Piriformis syndrome:1
		Gender: 30 F/10 M			Patient satisfaction:	New LBP:1
		Follow up: 12 months				Facet joint pain: 8
		Prior lumbar fusion: 30%				Trochanteric bursitis: 2
Cummings, 2013 [29]	18	Age: 64	3 or more positive provocative maneuvers, confirmatory joint injections	iFuse Implant System	Mean improvement in clinical outcomes:	Trochanteric bursitis 3
		Gender: 12 F/6 M			VAS -6.6pts,	Hematoma 1
		Prior lumbar fusion: 61%			ODI -37.5pts,	Fluid retention
					SF-12PCS 11.2,	1
					SF-12 MCS 20.4	Toe numbness 1
					Satisfaction:	implant malposition 1
					Very 55.6%, Somewhat 39%.	
					Would have surgery again: yes 83%, likely 6%	
Kibsgard, 2012 [34]	50, 28	Fusion (50 pts)	PSIS tenderness, positive straight leg raise, positive provocative maneuvers	Trans-iliac fusion or intra/extra-articular fusion between the ilium and the sacrum using cortical iliac window and iliac crest autograft.	Surgical patients after 1 year: 24 (48%) patients were good, 12 (24%) were fair, and 14 (28%) were poor.	Reoperation: 7
		Age: 58		Post-op care: In most cases the patients were confined to 6 weeks of bed rest.	No significant difference in ODI, VAS, or SF-36 between surgery and non-surgery patients after long-term follow-up.	Nonunion: 8
		Gender: 47 F/3 M				Jaundice: 1
		Follow-up: 23 yrs				Pulmonary embolism: 1
		Unilateral 21/Bilateral 25				Pin tract infection: 1
		Dx: Post-partum (30), Trauma (8), Idiopathic (12)				Complication rate: 20%
		Non-Surgery (28 pts)				Revision rate: 14%
		Age: 52				
		Gender: 28 F				
		Follow-up: 17 yrs				
Khurana, 2009 [22]	15	Age: 48.7 years	Tenderness over the posterior SI joint, positive provocative maneuvers, pain relief with SI joint block	10 mm Hollow Modular Anchorage Screw packed with demineralized bone matrix across the SI joint.	Blood loss: < 50 ml	None reported
		Gender 11 F/4 M		Post-op care: Partial weight bearing for six weeks and full weight bearing by 12 weeks.	LOS 2.7 days	
		Follow-up: 17 months			SF-36 increased: PF 37 to 80, GH 53 to 86	
		Unilateral 11/Bilateral 4			Majeed's: 37 to 79	
		Previous lumbar surgery: 40%			Good/Excellent: 13/15	
		Dx: Osteoarthritis (7), SI joint dysfunction(4), SI joint instability (3), Inflammatory Arthritis (1)			Fusion in all patients	
Al-Khayer2008 [23]	9	Age: 42 years	Tenderness over the sacral sulcus, positive provocative maneuvers,	10 mm Hollow Modular Anchorage Screw packed with demineralized bone matrix across the SI joint.	Blood loss: <50 ml	1 deep wound infection
		Gender: 9 F	X-rays to exclude other pain sources, relief from SI joint block	Post-op care: early mobilization w/in pain limits	No screw loosening, nonunion, or failure	Complication rate: 11%
		Follow-up: 40 mo			LOS: 6.9 days	
		Unilateral 6 /Bilateral 3			Return to work: 4/9	
		Symptom Duration: 30 mo			ODI decreased: 59 to 45	
		Prior treatments: Failed conservative treatment			VAS decreased: 8.1 to 4.6	
		Dx: Chronic SI joint pain			Satisfaction: 6.8 (out of 10)	
Wise, 2008 [24]	13	Age: 53 years	Relief with SI joint block	9 mm hole drilled through the longitudinal aspect of the SI joint. 2 cages packed with BMP placed across the anterior portion of the SI joint.	Blood loss: < 100 ml	Reoperation (nonunion): 1
		Gender: 12 F/1 M		Post-op care: limited waist bending, and a sacral belt for 6 mo; full activity at 6 mo	Length of stay: 1.7 days	Complication and Revision rate: 8%
		Follow-up: 29.5 mo			Fusion rate: 89%	
		Unilateral 7/Bilateral 6			Low back VAS improved 4.9 pts	
		Previous lumbosacral surgery: 8/13			Leg VAS improved 2.4 pts	
		Prior treatments: Failed > 6 mo of conservative therapy				
Buchowski, 2005 [21]	20	Age: 45 years	Sacral sulcus palpation,	Modified Smith-Petersen	Blood loss: 290 mL	Pseudoarthrosis: 3
		Gender: 17 F/3 M	positive provocative maneuvers,	Incision over posterior 2/3 of iliac crests. Graft stabilized w/ plate and screws.	Solid fusion: 17	Deep wound infection: 2
		Follow-up: 5.8 yrs	Pain relief with intraarticular SI joint injections	Post-op care: Non-weight bearing for at least 3 months.	LOS: 5.2 days	Painful hardware: 1
		Prior treatments: All failed nonoperative treatment			Return to work: 8/20	Revision surgery (anterior): 3
		Previous spine surgery: 15/20			SF-36 improved (except GH & MH)	Complication rate: 30%
		Symptom Duration: 2.6 yrs			AAOS MODEMS sig. improved (except Comorbidity)	Revision rate: 15%
		Dx: SI joint dysfunction (13), Osteoarthritis (5), Spondyloarthropathy (1), SI joint instability (1)			60% would have surgery again	
Giannikas, 2004 [35]	5	Age: 22 to 44 years	SI joint tenderness, positive provocative maneuvers, bone scan, relief with SI joint block	Two bone plugs harvested from the iliac crest and placed through the superior and inferior aspects of the SI Joint.	Complete pain relief: 4/5	None reported
		Gender: 3 F/2 M		Post-op care: Non-weight bearing for at least 3 months.	Partial pain relief: 1/5	
		Follow-up: 29 mo				
		Symptom Duration: 10 to 40 mo				
		Dx: Idiopathic (1), Previous trauma (4)				
Moore, 1997 [20]	77	Gender: 48 F/29 M	Relief with SI joint block	Modified Smith-Petersen technique with 15 cm incision to reveal the ilium and sacrum. Bone harvested from the ilium and placed in the SI joint after removing the cartilage. 2–3 cannulated screws to lock graft in place.	62/77 successful (80.5%)	Superficial wound infection: 1
		Unilateral 74/Bilateral 3		Post-op care: Non-weight bearing for 8 weeks.		Post-op radicular pain: 1
		Prior treatments: Failed 6 months of rehab programs				Sciatic notch fracture: 1
		Symptom duration: 6 to 84 mo				Pseudoarthrosis: 7
		Follow-up: 1 to 5 years				Complication rate: 13%
		Dx: Chronic painful dysfunction				
Keating, 1995 [19]	26	Age: 38.3 years	Relief with SI joint block	Inferior SI joint debrided, decorticated, and packed with bone graft. Secured with 2 lateral compression screws.	Pain decreased: 6.1 to 2.9	None reported
		Follow-up: 16 weeks		Post-op care: 16 week rehabilitation program.	Work Status increased: 2.3 to 3.3	
		Prior treatments: Failed 6 weeks of aggressive rehab			5 patients returned to work after 16 mo of unemployment	
		Symptom duration: 38.3 mo				
		Dx: Chronic LBP				
Waisbrod, 1987 [17]	21	Age: 42	Tenderness over the SI joint, positive provocative maneuvers, pain provocation w/ NaCL injection, relief w/ SI joint block	SI joint excised and packed w/ iliac crest bone graft and ceramic blocks.	11/21 Satisfactory results	Pseudoarthrosis: 2
		Gender: 18 F/3 M		Post-op care: Spica cast for 8 weeks.		Infection: 1
		Follow-up: 30 mo				Complication rate: 14%
		Previous spine surgery: 7/21				
		Symptom duration: > 2 years				
		Dx: SI joint pain				

Pain and degeneration of the SI joint after lumbar spinal fusion are common occurrences, with up to 43% of these patients experiencing SI joint pain and 75% showing radiographic changes [6-8]. Open surgical techniques, whether using a posterior or anterior approach, show that these patients experience poorer outcomes [7]. Mason et al. found outcomes in patients treated with prior lumbar fusion to be significantly diminished after MIS SI joint fusion using hollow modular anchorage screws [36]. In contrast, three studies that report no difference in outcomes for patients with and without prior lumbar fusion using the MIS technique reported herein [26,28,29].

To our knowledge, this is the first multi-center comparison of open and MIS SI joint fusion surgery. Rigorous statistical methods (age- and gender-matching as well as controlling for a history of prior lumbar fusion) were employed in an attempt to account for patient characteristics that could affect outcomes after SI joint surgery. Results of this study illustrate the advantages expected of MIS spinal surgery: reduced surgically induced tissue damage, blood loss and surgical morbidity, and length of hospitalization. These parameters have been implicated as risk factors for surgical site infections [37-39]. Additionally, patients treated with MIS SI joint fusion experienced significantly greater improvements in pain than those who underwent open surgery, regardless of history of prior lumbar spinal fusion. The triangular shape, interference fit and TPS coating of the MIS implant allows for both immediate stabilization and long-term biological fixation of the device. Pseudoarthrosis, screw loosening, and spinal implant irritation were sources of surgical revisions (43%) in the open surgery cohort. None of these complications were present in the MIS cohort. Revisions (3.5%) in the MIS group were the result of suboptimal implant placement.

This study is not without limitations. First and foremost, it is not a randomized prospective trial. As discussed by McAfee et al., randomizing patients is extremely challenging in today’s postmarket environment [40]. Many patients are unwilling to be randomized to a more invasive surgical procedure when a newer minimally invasive technique procedure is available without participating in such a clinical trial. Such trials have even been denied by IRBs, who contend that the benefits of MIS surgery (typically shorter operating time, less blood loss and shorter hospital stay) make it unethical to subject patients to randomization [40].

This study is also not concurrent. Most surgeons interviewed during the site identification process performed either open *or* MIS SI joint procedures, but not both. In an incidence rate study of data obtained from the AMA/Specialty Society Relative Value Scale Update Committee (RUC) database, less than 200 open SI joint fusion procedures were performed annually between 2001 and 2008 [41]. In 2012, 85% of all SI joint fusions performed were minimally invasive. It is apparent that the open surgical technique has fallen out of favor with the introduction of MIS methods.

Whether our results are reflective of the entire population of patients who undergo open or MIS fusion is not known. The low rate of open SI joint fusions performed annually coupled with the rapid adoption of MIS techniques with favorable safety and effectiveness profiles makes a prospective randomized controlled trial highly unlikely.

The current study also lacks patient-reported outcomes typically collected in controlled trials, such as Oswestry Disability Index and SF-12 or SF-36. These outcomes instruments are labor intensive for both patients as well as office staff, hence surgeons in private practice do not typically collect them. Not all sites had complete data sets to include EBL, surgical operating time and length of stay for every patient. Finally, pain rating questionnaires, included as part of each surgeon’s standard practice, may have varied in content and been administered differently across sites.

## Conclusion

Minimally invasive SI joint fusion using a series of triangular, titanium, TPS coated implants (iFuse Implant System) results in more favorable perioperative measures, fewer reoperations and significantly improved clinical outcomes compared to traditional open surgical SI joint arthrodesis.

## Competing interests

RC and DC are employees of SI-BONE, Inc. AGS, LR, DS, MG and NS are paid consultants of SI-BONE, Inc. LR and NS are stockholders in SI-BONE, Inc. JK, MM have no competing interests.

## Authors’ contributions

AGS, LR, DS, MG, JK, MM, NS performed patient surgeries, and contributed to manuscript draft. RC and DC drafted manuscript, performed data analysis and interpretation. All authors reviewed and provided final approval of manuscript.
